# Can apparent resting state connectivity arise from systemic fluctuations?

**DOI:** 10.3389/fnhum.2015.00285

**Published:** 2015-05-15

**Authors:** Yunjie Tong, Lia M. Hocke, Xiaoying Fan, Amy C. Janes, Blaise deB Frederick

**Affiliations:** ^1^McLean Imaging Center, McLean HospitalBelmont, MA, USA; ^2^Department of Psychiatry, Harvard University Medical SchoolBoston, MA, USA; ^3^Department of Biomedical Engineering, Tufts UniversityMedford, MA, USA

**Keywords:** resting state networks, BOLD fMRI, cerebral blood flow, slow oscillations, systemic oscillations

## Abstract

It is widely accepted that the fluctuations in resting state blood oxygenation level dependent (BOLD) functional MRI (fMRI) reflect baseline neuronal activation through neurovascular coupling; this data is used to infer functional connectivity in the human brain during rest. Consistent activation patterns, i.e., resting state networks (RSN) are seen across groups, conditions, and even species. In this study, we show that some of these patterns can also be generated from the dynamic, systemic, non-neuronal physiological low frequency oscillations (sLFOs) in the BOLD signal alone. We have previously used multimodal imaging to demonstrate the wide presence of the same sLFOs in the brain (BOLD) and periphery with different time delays. This study shows that these sLFOs from BOLD signals alone can give rise to stable spatial patterns, which can be detected during resting state analyses. We generated synthetic resting state data for 11 subjects based only on subject-specific, dynamic sLFO information obtained from resting state data using concurrent peripheral optical imaging or a novel recursive procedure. We compared the results obtained by performing a group independent component analysis (ICA) on this synthetic data (i.e., the result from simulation) to the results obtained from analysis of the real data. ICA detected most of the eight well-known RSNs, including visual, motor, and default mode networks (DMNs), in both the real and the synthetic data sets. These findings suggest that RSNs may reflect, to some extent, vascular anatomy associated with systemic fluctuations, rather than neuronal connectivity.

## Introduction

The use of resting state functional MRI (rsfMRI) to study baseline brain activation in the absence of external stimuli has increased dramatically in recent years (Biswal et al., 1995; Greicius et al., 2003; Fox et al., 2005; Damoiseaux et al., 2006). Resting state network (RSN) analysis is based on the premise that information is exchanged between functionally related brain regions continuously, even during rest, leading to correlated neuronal activations among these regions in resting state. These correlated neuronal activations in turn lead to coherent spontaneous low frequency (~0.1 Hz) fluctuations in blood oxygen level dependent (BOLD) signals through neurovascular coupling.

Implicit in these analyses are a number of assumptions that are not frequently articulated. The first is that the hemodynamic response to spontaneous neuronal activations is the main source of spatially correlated low frequency oscillations observed in the BOLD signal. The second is that within each RSN, synchronized neuronal activations lead to high temporal correlations between the BOLD signals from these functionally related regions. Finally, it is generally assumed that the particular pattern of coupled spontaneous neuronal activations is unique to each RSN, leading to different timecourses for each of the networks. Therefore, we are able to identify different RSNs using correlation analysis, in which we search for the regions that share the same low frequency BOLD signals, either using a seed region placed in an area of interest (Biswal et al., 1995; Hampson et al., 2002) (i.e., seed analysis), or a data-driven independent component analysis (ICA) (Calhoun et al., 2001; Beckmann et al., 2005), where independent spatiotemporal RSNs can be derived simultaneously without a predetermined regressor.

However, as we know, the BOLD signal is not a direct measurement of neuronal activation, but rather a measurement of changes in blood flow, oxygenation, and volume (Obrig et al., 2000; Wise et al., 2004; Birn et al., 2006; Tong and Frederick, 2012). While these changes can be, and are, caused by neuronal activation through neurovascular coupling, they can also arise from any other physiological processes that affect blood oxygenation or volume. In our recent resting state studies, we have found that a significant portion of the slow oscillations (~0.1 Hz) observed in BOLD fMRI is closely associated with the propagation of systemic low frequency oscillations (sLFOs) through the vasculature (Tong and Frederick, 2010, 2014). The reason these sLFOs are called “systemic” is that the same signals found propagating through the brain, are found in the periphery (e.g., fingertips and toes) as well, when using simultaneous near infrared spectroscopy (NIRS) recordings (Tong and Frederick, 2012; Tong et al., 2013). Although the origins and functions of these sLFOs are not known (Sassaroli et al., 2012), it is clear that temporally shifted versions of this signal are widely present in the BOLD signal of the majority of the voxels in the brain. They seem to travel with the blood and reach different voxels at different time delays determined by the path blood takes through the cerebral vasculature.

Brain regions with similar vascular time delays will have high temporal correlations, since the sLFOs will arrive in these regions at the same time. Because of the high symmetry in the cerebral vasculature, these correlated regions will tend to be symmetric. Blood takes ~6–9 s to travel from the carotid arteries to the jugular veins through which it leaves the head (Crandell et al., 1973; Schreiber et al., 2002; Tong and Frederick, 2010), so for any time lag within this range, there will be a pattern of spatial voxels sharing a timecourse at that delay time. This is very different from patterns derived from spontaneous neuronal activities, in which high correlations between regions mean that the regions share a signal derived from the unique neuronal fluctuations for each network. However, correlation-based RSN analyses might not be able to differentiate the sLFOs related correlations from neuronal ones.

In this study, we examine to what extent apparent connectivity may arise from these non-neuronal sLFOs alone, using commonly used RSN analyses, such as ICA and seed analysis. We focus primarily on ICA analysis here, as we feel that ICA's sensitivity toward temporal shifts in the BOLD signals, and how that sensitivity affects these analyses, is not widely known.

To determine whether time shifted systemic signals can result in apparent RSNs, we must answer three questions.

1. What does ICA do when presented with image data consisting of multiple regions with the same timecourse, differing only in the temporal shift of the timecourse? In other words, could ICA derive multiple different networks from moving sLFOs alone? To answer this question, we applied ICA on synthetic BOLD data consisting of regions with the same timecourse over the range of time delays (−2 to +2 s, about the range of blood arrival times observed in the majority of the brain *in vivo*), to see if separate networks were generated.
2. What is the distribution (amplitude and delay) of sLFO signals seen in real subjects' task free data? In other words, can we quantify the parameters of the sLFO signals in each brain voxel? We have addressed this question in our previous work (Tong and Frederick, 2014) and will discuss it briefly in the methods. In short, we are able to accurately calculate the arrival time and amplitude of sLFOs at each relevant voxels in resting state data using two different methods.
3. What kind of networks would be generated from these moving sLFOs alone using common methods, in the absence of any neuronal signals? Are these networks of sLFOs similar to the well-known RSNs? To assess this problem, we performed a group analyses (seed based and ICA) on synthetic BOLD data (where each voxel has an identical timecourse, delayed by the voxel-specific temporal delays of the sLFOs calculated from the real data). These synthetic data only reflect the subject-specific propagating information of these sLFOs in the brain, not neuronal signals. The resulting “networks” were compared with well-known RSN templates as well as RSNs derived from the real resting state BOLD data of the same group of subjects.

## Materials and methods

### Protocol

fMRI resting state studies were conducted in 11 healthy participants (6 M, 5 F, average age ± SD, 31.3 ± 11.7 years). In the resting state studies, participants were asked to lie quietly in the scanner with their eyes open and view a gray screen with a fixation point in the center. The resting state scans lasted 360 s for 11 participants. The Institutional Review Board at McLean Hospital approved the protocol and informed consent from was signed by every participant before the experiment.

All MR data were acquired on a Siemens TIM Trio 3T scanner (Siemens Medical Systems, Malvern, PA) using a 32-channel phased array head matrix coil. After acquiring a high resolution localizer image, (MPRAGE, TR/TI/TE = 2530/1100/3.31, 256 × 256 × 128 voxels over a 256 × 256 × 170 mm sagittal slab, GRAPPA factor of 2), multiband EPI (University of Minnesota sequence cmrr_mbep2d_bold R008) (Moeller et al., 2010; Xu et al., 2013) data was obtained with the following parameters: (TR/TE = 400/30 ms, flip angle 43°, matrix = 64 × 64 on a 220 × 220 mm^2^ FOV, multiband factor = 6, 30, 3.0 mm slices with 0.5 mm gap parallel to the AC–PC (anterior commissure–posterior commissure) line extending down from the top of the brain. Concurrently, a MRI-compatible optical NIRS probe (1.5 cm separation between collection and illumination fibers) was placed over the tip of the left middle finger. NIRS data was recorded continuously before, during and after the resting state fMRI acquisition with an ISS Imagent (ISS, Inc., Champaign, IL) at 690 and 830 nm with 12.5–25 Hz acquisition rate.

### Analytical method

For each participant, the standard fMRI preprocessing steps, including brain extraction, motion correction, slice-time correction, smoothing (5 mm) were applied to the original BOLD signals (using FEAT v6.00 of FSL 5.0) (Jenkinson et al., 2012). We then applied a Fourier domain bandpass filter (0.01–0.2 Hz) in MATLAB (The Mathworks, Natick, MA) on all the resulting data to remove the high frequency physiological signals of respiration and cardiac pulsation from the BOLD data (which was possible due to the ultrafast fMRI acquisition).

#### Simulations – ICA

**Figure 1:**
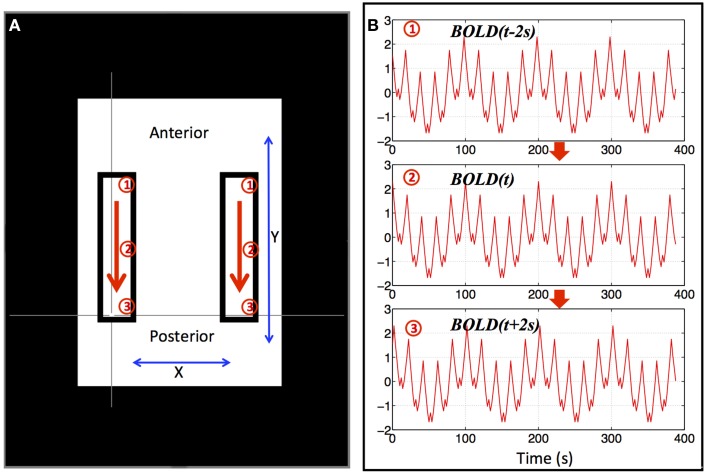
**Synthetic data for a single “subject.”** Two identical blocks were selected, as shown in **(A)**, in which, artificial traveling waves (as shown in **B**) were used to replace the original BOLD signals. The red arrows indicate the direction of the traveling wave and synthetic data from three positions marked by circled numbers (1–3) were shown in **(B)**. The remaining BOLD signal was also replaced by an identical synthetic data as (2) in **(B)**.

Since the signal power of the resting state BOLD data is mainly in the low frequency range, we used a combination of multiple sinusoid waves of different low frequencies (0.01–0.2 Hz) to simulate a basic BOLD fMRI timecourse (as shown in Equation 1). Moreover, we generated synthetic propagating sLFO waves in two symmetrical regions (rectangular) as shown in Figure 1 by progressively delaying this timecourse in the *y* direction. The propagating wave started at the anterior portion of these two areas and gradually “moved” toward the posterior (as indicated by the arrows). To reflect this, we used the BOLD signal (as in Equation 1) with gradually changing Δ*t* (***BOLD***(*t* + Δ*t*)) to simulate the BOLD signals in these areas [Δ*t* gradually changed from −2 to +2 s as the wave moved from anterior to posterior within the areas (see Figure 1)]. The 4 s range of propagation delays was selected based on the mean transit time of the cerebral blood flow in the brain seen in previous studies (Crandell et al., 1973; Schreiber et al., 2002). The TR in the synthetic dataset is 2 s and a total of 195 volumes were calculated. To match properties of real fMRI data, the synthetic BOLD signal of each voxel had a mean of 10^4^ and a standard deviation of 4% of the mean. White noise with a standard deviation of 1% of the mean was added to each voxel to simulate scanner noise.

(1) $$\begin{array}{l} BOLD(t)=0.8\times \text{sin }\left(0.01\cdot 2\pi \cdot t\right)+1\times \text{sin }\left(0.05\cdot 2\pi \cdot t\right)\\ \;+0.3\times \text{sin }\left(0.1\cdot 2\pi \cdot t\right)+0.2\times \text{sin }\left(0.2\cdot 2\pi \cdot t\right)\\ \;+0.2\times \varepsilon (t); \end{array}$$

**ε(t)** is the white random noise generated by a MATLAB function (random).

We applied Melodic ICA (Multivariate Exploratory Linear Optimized Decomposition into Independent Components (Melodic 3.10) (Beckmann and Smith, 2004; Beckmann et al., 2005) from FSL (Smith et al., 2004; Jenkinson et al., 2012) on this single subject's synthetic data with spatial smoothing (5 mm), variance normalization and a fixed dimensionality of 10 components.

#### Group ICA on all subjects

##### Resting state analysis – group ICA on real data

We performed a group MELODIC ICA analysis on all 11 participants' real resting state data with the following setups: spatial smoothing (5 mm), variance-normalize timecourses, multi-session temporal concatenation. The dimensionality in MELODIC was set to 30, a number routinely used for ICA studies (Beckmann and Smith, 2004). The final results were all registered to the MNI152 2 mm^3^ standard space template (Montreal Neurological Institute, Montreal, QC, Canada).

##### Resting state analysis – group ICA on synthetic data

The analysis of the previous section was repeated on synthetic datasets with no neuronal information. To generate the synthetic data, we first calculated the 3D sLFO delay map from each subject's own resting state data. The delay map (3D image) reflects the subject-specific propagation of the sLFOs throughout the brain. The value of the voxel in a delay map marks the relative arrival time of the sLFOs at this voxel. For instance, a value of 2.0 means that the sLFOs arrived at this voxel 2 s later than the time arrival time at a reference voxel. We then generated synthetic systemic BOLD data (using the timecourse shown in Equation 1) for each voxel, where the same timecourse, representing the systemic signal, is used in every voxel; in each voxel the temporal shift of this timecourse is determined by the delay value for that voxel and scaled to the local BOLD signal intensity. For instance, the synthetic timecourse will be shifted by 2 s in the voxel, for which the delay value is 2 and then the whole timecourse will be rescaled to the intensity of the original BOLD. NB: the timecourse itself is arbitrary – the only important characteristic is that the signal is bandlimited to the low frequency range (0.01–0.2 Hz). Using sums of sinusoids, filtered white noise, or the subject's actual fingertip NIRS signal yields the same results.

In order to ensure that the delay map estimation was not in some way contaminated by neuronal signals, the delay maps were generated twice using two independent procedures. The first procedure used was a data-driven recursive method. The details of the method can be found in our previous publication (Tong and Frederick, 2014). In short, the data-driven method employs a recursive procedure to extract sLFOs from the BOLD signals of each subject. The procedure starts with seed selection. Since we believe these sLFOs are related to the blood signal, the seed is chosen from a blood vessel voxel (e.g., in the Superior Sagittal Sinus) to avoid neuronal activation. The timecourse of the seed is extracted and used as a regressor to cross-correlate with all the BOLD signals from the rest of the voxels. Voxels were selected that met the following conditions: (1) the highest correlation is greater than 0.5; (2) the time lag of this highest correlation is 1 (or −1, depending on the direction of the flow we are tracking. Unit:TR).

The averaged timecourse of these voxels becomes the new “seed” regressor and the procedure is repeated. Every new regressor is similar to the previous regressor (high correlation) while being temporally shifted by one TR, representing sLFO signals that have moved in space compared to the voxels detected by the previous regressor (an example of these regressors is depicted in Supplementary Figure 1). This set of regressors is then used to assess the arrival time of sLFOs at relevant voxels statistically by using FSL FEAT (see details in Tong and Frederick, 2014). The temporal delay was derived only from those voxels having significant correlations (*z* > 6) with one of these regressors. This is critical to guarantee the delay time calculated is from the sLFOs, not from other signals in the BOLD. In the final delay-map, the value in each voxel represents the relative arrival time of these sLFOs at the specific voxel. We synthesized the data using the delay maps for all 11 subjects.

In the second procedure, we used our previously described method (Tong et al., 2011), in which the oxyhemoglobin concentration timecourse measured by NIRS in the periphery is used as a systematic physiological noise regressor. We first converted the raw optical data into the changes in oxy-hemoglobin concentration (Δ[HbO]) and the changes in deoxy-hemoglobin concentration (Δ[Hb]) (Matcher et al., 1994). We extracted the corresponding timecourse of Δ[HbO] (over the time range matching the fMRI scan) and downsampled it to the fMRI TR (0.4 s). The voxel-wise cross correlation with this timecourse was calculated. For each voxel, the temporal shift corresponding to the maximum correlation coefficient (threshold is *R* = 0.15) is used as the temporal delay of that voxel. Δ[HbO] is used instead of Δ[Hb] because of its significantly higher signal-to-noise ratio (SNR) (Zhang et al., 2010b; Niu et al., 2011) due to it's higher concentration (the oxygen saturation of the blood in the periphery is typically over 95%). After obtaining the delay-map, the synthetic data of 11 subjects was generated using the procedure described above.

Once the synthetic datasets were constructed, group MELODIC analyses with the same parameters used in the previous analysis of real data were performed on each set of synthetic data. The results were compared with a widely used RSN template (Beckmann et al., 2005). For a fair comparison of the results from the real and synthetic data (both were put through the same procedures), we rescaled the synthetic data by the mean and amplitude of the corresponding real BOLD signal, leaving the temporal oscillation the only distinct variable. The scaling effect will not affect the result since the variances have been normalized in the process (i.e., variance–normalize).

#### Seed analysis of real and synthetic data (group)

In addition to the ICA analysis, the real and synthetic data underwent a seed analysis. The default mode network (DMN) was selected due to its robustness. The DMN seed was a 10 mm spherical region of interest in the posterior cingulate cortex (Watanabe et al., 2013) centered at the MNI coordinates 45, 35, and 49. The average timecourse across all voxels within the region of interest were extracted for every individual participant. The resulting timecourses were used as regressors in a subject-specific general linear model (FSL Feat – Lower level analysis) to define the DMN. A second-level mixed-effects analysis (FSL Feat – Higher level analysis) was subsequently run to define the average DMN across all subjects. At the group level, statistics were corrected for multiple comparisons using a cluster threshold of *z* = 2.3, *p* < 0.05.

## Results

**Figure 2:**
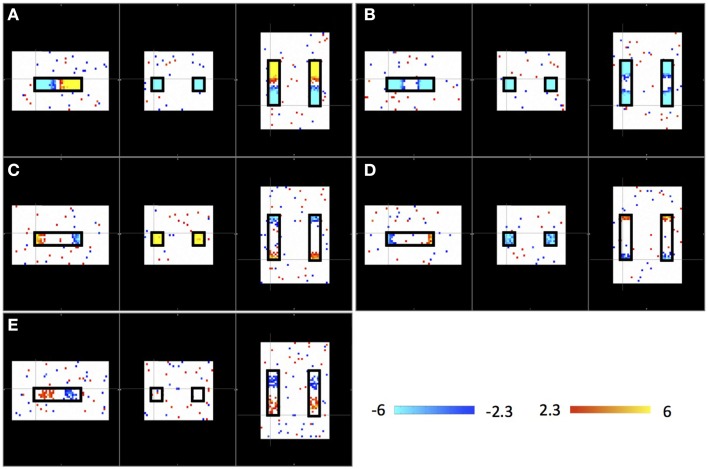
**Five ICs **(A–E)** resulting from ICA on a single “subject.”** Each IC pattern is shown in three viewpoints (Sagittal, Coronal, and Axial). The locations of synthetic traveling waves are indicated by the black boxes in all three views. The color bar represents the *z*-value as result of ICA.

**Figure 3:**
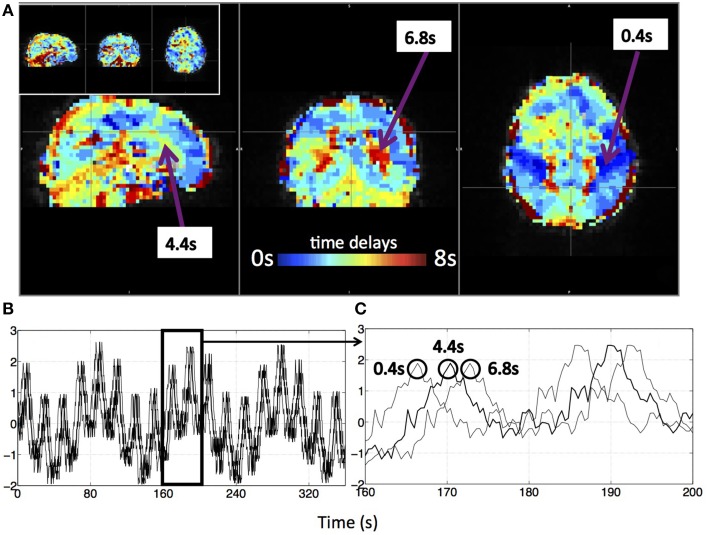
**A delay map of the synthetic data of one subject derived with the recursive method (A) and its synthetic BOLD data (B,C)**. Three example voxels were selected from the delay map with their time delays **(A)** and the corresponding synthetic temporal traces were shown in **(B)**. **(C)** The enlarged section of **(B)** with circles indicating the corresponding voxel of the marked trace. The delay map derived with the NIRS method of the same subject is shown as an inlet in **(A)**.

Figure 2 shows the results of an ICA of the synthetic data from a single “subject” where a single arbitrary timecourse is given a time shift proportional to its vertical position within the highlighted box (as shown in Figure 1). Among 10 resulting independent components (IC), five IC patterns related to the artificially moving sLFO waves were identified, including the patterns: (1) associated with large, intermediate, and small sizes (as shown in Figure 2A, Figure 2B, and Figures 2C,D, respectively); (2) associated with different locations inside the highlighted box (as shown in Figures 2A,C,E); (3) associated with uniform (in Figures 2A,B) or scattered distribution as shown in Figure 2E. All the patterns are symmetric.

**Figure 4:**
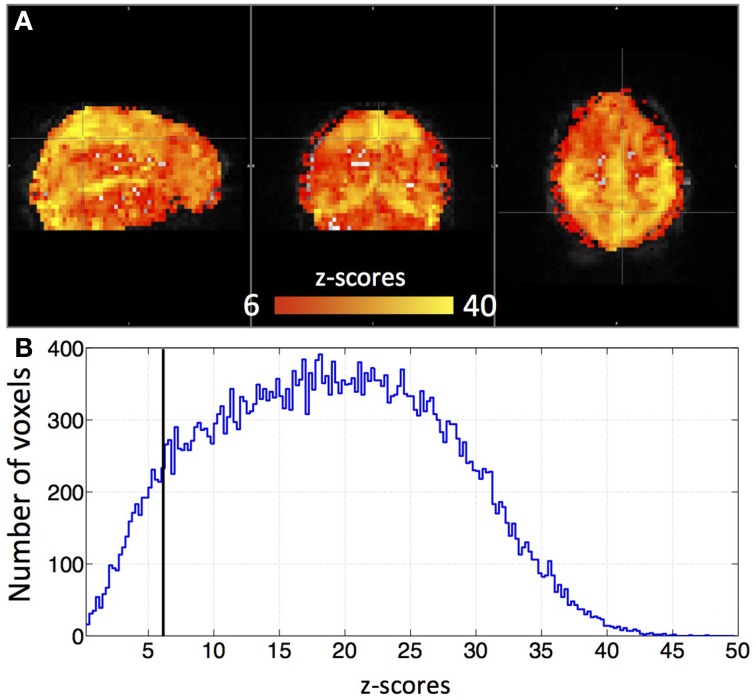
**A maximum *z*-statistic map (A) of the same subject as in Figure 3**. The histogram of the maximum *z*-statistic map is shown in **(B)**, in which, the black line (*z* = 6) indicates the threshold.

Figure 3 shows an example delay map of one subject derived using the recursive method, together with the temporal delays of three example voxels and their synthetic timecourses. In addition, a similar delay map (of the same subject) derived from the NIRS method is shown as an inset in Figure 3A (upper left). The following observations can be made: First, the variations of the colors in the map indicate that these sLFOs do reach different voxels at different times. The overall systematic color patterns reflect the underlying cerebral blood vasculature. For example, the longer delays (shown as red-yellow in Figure 3) happen at the draining veins (i.e., Superior Sagittal Sinus, straight sinus). Second, the time it takes a wave to propagate through the entire brain is about 7 s, which is consistent with the mean transit time of cerebral blood flow (Crandell et al., 1973). Lastly, the draining system (with clearly identifiable veins) is more prominent in the map than the arteries are. This is because BOLD fMRI is more sensitive to veins than arteries (Menon, 2012). To demonstrate the significance of the BOLD signal correlation with these sLFO regressors, we showed the maximum *z*-statistic map of the same subject in Figure 4A. The maximum *z*-value at each voxel reflects the significance of the correlation between the BOLD signal of that voxel and the corresponding recursive regressor. From Figure 4A, we can observe that most of the BOLD signal is significantly correlated with one of these regressors (max *z* > 6). This significance threshold is more stringent than Bonferroni correction. The most significant *z*-values (max *z* > 30) are found in the gray matter as well as large draining veins. Figure 4B shows the corresponding histogram of the maximum *z*-statistic map, which further demonstrates the significant correlations between sLFOs and BOLD and the wide spatial extent of the sLFOs in the BOLD signals.

**Figure 5:**
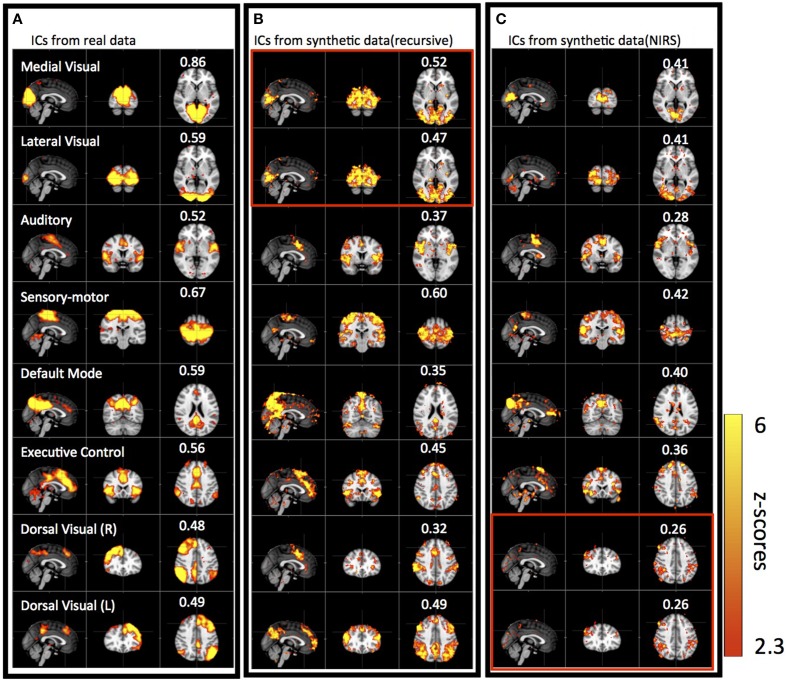
**Eight groups of ICs resulting from group ICA on 11 subjects' real BOLD data (A), synthetic BOLD data derived with the recursive method (B), and synthetic BOLD data derived from NIRS data (C)**. The eight ICs were selected in each group result (real vs. synthetic) using RSNs templates (Beckmann et al., 2005) and the value in each IC shows the spatial correlation coefficient calculated between that IC and the corresponding RSN from the template. The ICs in the red block are the same IC.

Figure 5 shows results of three group ICAs: using actual resting state data (Figure 5A); using the synthetic data derived from the recursive method (Figure 5B); and using the synthetic data derived from NIRS method (Figure 5C). The eight spatial patterns were selected from each ICA result by spatial correlation (fslcc in fsl, threshold *R* > 0.25) using a well-known RSN template (Beckmann et al., 2005), including Medial Visual (45, 19, 37 coordinates in MNI152 2 mm^3^ standard space), Lateral Visual (45, 19, 37), Auditory (45, 65, 35), Sensory Motor (45, 51, 68), Default-mode (45, 32, 47), Executive-control (45, 73, 56), Dorsal Visual (Right, 45, 78, 56) and Dorsal Visual (Left, 45, 78, 56) network. The corresponding ICs were listed in horizontal groups with their corresponding spatial correlation values (calculated with RSN templates) for easy comparison. IC patterns matching well-known RSNs were found with both types of synthetic as well as in the real data. The spatial correlation coefficients with the RSN templates were highest for the real data (*R* = 0.48–0.86), decreased slightly with the synthetic data derived with the recursive method (*R* = 0.32–0.6), and reduced further in the synthetic data derived with the NIRS method (*R* = 0.26–0.42), while still being above the threshold (*R* = 0.25). Among the ICs from the synthetic data, the ones resembling the Medial, Lateral Visual networks, the Sensory Motor network, the Executive Control network and the Dorsal Visual (L) network have the highest correlation coefficients (*R* > 0.4). Medial, Lateral Visual networks cannot be individually identified in the results derived from the synthetic data using the recursive method (marked in red box in Figure 5B), while Dorsal Visual networks (L,R) cannot be separated in the results derived from NIRS method (marked in red box in Figure 5C). The asymmetry in RSN patterns [i.e., Dorsal Visual networks (L,R)] detected in the real data, as in Figure 5A, are replaced with the symmetrical ones in the results derived from the synthetic data using the recursive method (Figure 5B). Interestingly, unsymmetrical patterns [i.e., Dorsal Visual (L,R)] was detected in the results derived from the NIRS method (last two in Figure 5C).

**Figure 6:**
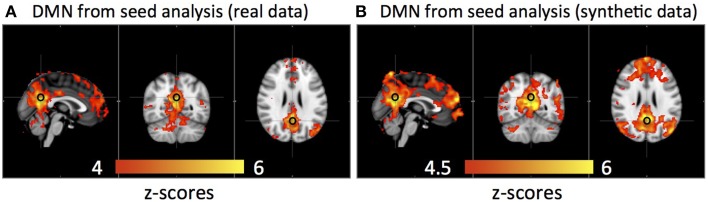
**Averaged default mode network detected by seed analysis from 11 subjects' real resting state data (A) and synthetic resting state data (B)**. Black circles indicate the seed location.

Figure 6 shows that the DMN was detected from the same group of subjects using both real data and synthetic data by seed analyses (seed location is marked by a black circle). The spatial correlation between these two results is *R* = 0.83.

## Discussion

### ICA is highly sensitive to temporal shifts in bold signal

In this study, we show that the ICA method used in many resting state analyses is sensitive to the temporal shifts in the BOLD signals. The same temporal signal, shifted a few seconds in one direction, can give rise to multiple distinct spatial networks. As shown in Figure 2, five symmetric “networks” are found when the only difference in the voxels is the time shift of an identical signal over a 4 s range. This has somewhat important implications for RSN studies, since the following two conditions: (1) time shifts in the signals; and (2) symmetric distribution of these signals with certain time shifts, are characteristic of the propagating sLFOs, which are prominent in the resting state data of real subjects, as mentioned in the introduction. The concern is that many symmetric artificial networks can be “detected” by ICA along the cerebral blood flow path, even without neuronal contribution. On the same synthetic data set, we tried different time shifts range (instead of 4 s) and found ICA could not separate ICs when the time range is below 0.3 s. This is an empirical result and more theoretical studies are needed to understand the effects of temporal shifts on ICA.

### The sLFO delay maps can be derived from resting state data

To test this hypothesis on real subjects, we generated synthetic resting state data. The first step was to calculate the subject-specific delay maps from 11 healthy subjects' resting state data. We did this using two different methods.

First, we employed a recursive method that takes advantage of the ultrafast fMRI (TR = 0.4 s) acquisition. The method, which we have previously described in detail (Tong and Frederick, 2014), is designed specifically to accurately assess the temporal shifts between sLFOs from relevant BOLD signals in the brain using resting state data, and avoid neuronal activity. The advantages of this method are: (1) the sLFOs and delay map can be derived directly from fMRI data itself; (2) the procedure is very robust, resulting in similar delay maps regardless of the initial seed selection. However, the method might also be prone to the influence of the global neuronal signals, which we discuss in detail in The Delay Maps Were Calculated to Only Reflect the Temporal Delays of the sLFOs in BOLD Signals.

For the second method, we utilized the fact that sLFOs travel throughout the body (Tong et al., 2012) to calculate the delay map in the brain using the simultaneously recorded NIRS Δ[HbO] at the fingertip. This method is very straightforward, and is likely to give quite accurate delay values. However, there are a few factors, which may reduce the sensitivity of the technique. The NIRS peripheral data catches the essential sLFOs, but it has following caveats: (1) It measures a single point that is far from the brain, which may add noise to the correlation, due to peripheral vascular effects; (2) it is the Δ[HbO] signal, not the direct BOLD signal, so the signal waveform may not exactly match that of the BOLD; (3) it is a static signal, and does not reflect amplitude changes of sLFOs as they propagate through the brain. These may affect its sensitivity in calculating the delay map. However, these delay maps are very straightforward to calculate, and are unequivocally independent of any neuronal signal (these delay maps use a wholly extracerebral signal in calculating the time delays).

As shown in previous studies (Tong and Frederick, 2014), we have demonstrated the dynamic flow patterns obtained from these two methods likely reflect the cerebral blood flow in terms of dynamic spatial distribution and temporal duration (see the Supplementary Movie). This has not yet been fully validated (dynamic susceptibility contrast MRI experiments are being conducted to validate this hypothesis). However, the fact that similar patterns have been derived among subjects using multiple methods demonstrates the robustness of the sLFO effect, indicating its universal existence in the resting state data.

### The delay maps were calculated to only reflect the temporal delays of the sLFOs in bold signals

There are two concerns in calculating these delay maps. One concern is that these delay maps can be biased by the regional spontaneous neuronal signals. Even more, we might introduce some artificial delays, essentially filtering the low frequency fMRI data to the frequency and phase profile of the sLFOs (or of even random timeseries), which were not there in the first place. This is unlikely for the following reasons: (1) these physiological regressors (i.e., sLFOs) are obtained or derived from actual data recorded in the periphery or brain, which have been shown to be highly correlated with the BOLD of the resting state—they are not random, non-related low frequency oscillations; (2) the voxel-wise *z*-value calculated by these real physiological regressors (using GLM) are statistically significant, as shown in Figure 4A. Figure 4B shows the histogram of these *z*-values. From these graphs, we observe that the mean *z*-value calculated at each voxels using the recursive regressors is around 20 (which is the case for all the subjects). As the delay values are only calculated from the voxels that significantly correlated with these regressors (*z* > 6), we can ensure that the corresponding delay values accurately reflect only the arrival time of these sLFOs, no other signals. On the contrary, correlation with random signals do not generate maps with statistical significance; (3) if the spontaneous neuronal signals do affect the delay map, they should influence the delay map differently across the subjects due to its spontaneous nature. However, the delay map and dynamic patterns derived from different subjects are not random. They mimic the cerebral flow patterns (as shown in the Supplementary Movie) both in spatial pattern and delay times and are very consistent between subjects. A *z*-statistic map and its corresponding delay map of another subject were shown as Supplementary Figure 2, in which, similar patterns as in Figure 3 (and Figure 4) can be observed. Lastly, we have generated synthetic delay maps with randomly assigned delay values, which did not produce a single meaningful RSN. In short, the delay maps are inherent properties of the resting state data. The estimation methods expose these properties, but do not create them.

The other concern is the possibility that some global neuronal activations might influence the delay map (Olbrich et al., 2009; Scholvinck et al., 2010; Tal et al., 2013). However, this is unlikely for the recursive method. First, the evolution of the regressors is very slow (as shown in Supplementary Figure 1). Any two regressors, with seconds of temporal delays between them, are almost identical to each other. The global neuronal signals would evolve much faster. Second, the dynamic patterns derived as result of these regressors and delay map are similar to the cerebral blood flow pattern (as shown as Supplementary Movie) and last about 7–8 s, which are also too long for the global neuronal signals. Moreover, the ICA with the synthetic data did not produce only one IC resembling a RSN, which would be a reasonable result if a small neuronal signal were included in the method. Many RSN-resembling ICs were found through this method, strongly indicating that the timecourses of these networks are correlated, with phase delays of several seconds, which is not consistent with the features of global neuronal activity. Lastly, it is even less likely that neuronal activity affects the delay maps derived from NIRS data. The sLFO regressors in this case are all extracted from the periphery (i.e., fingertip), where there is presumably no neuronal contamination. Yet, almost all the corresponding ICs (eight from the template) with high spatial correlation (fslcc > 0.25) were detected in the result. Among these ICs, the IC with the asymmetric pattern is particularly interesting [pattern 7 in Figure 5A (Dorsal Visual (R))]. As we know, these patterns could not result from a totally symmetric delay map. We do not fully understand why the delay map from NIRS data is asymmetrical, which led to the asymmetric ICs result. The likely reason is that sLFOs do have small arrival time differences between the two hemispheres (perhaps due to slight differences in the anatomy of the two carotids); however, the recursive procedure suppresses this difference by averaging many voxels to get the next regressor, while the procedure using NIRS data did not.

In summary, the most critical facts we demonstrate are that (1) there are robust internal systemic correlations between BOLD timecourses throughout the brain with time delays of up to several seconds, which are unlikely to be the result of spontaneous neuronal activations; (2) these temporal shifts between sLFOs at relevant voxels are present even when using very different derivation methods, so they are unlikely to be due to an artifact of the processing methodology.

### Networks derived from sLFOs are similar to the RSNs

The ICA results of the two synthetic datasets (from both the recursive and NIRS methods) show high similarity with those from the real data, as shown in Figure 5. Here we would like to remind the reader that in the synthetic data used here, the only subject specific (i.e., “real”) information is the delay map, which provides the time shifts applied to the identical sinusoid waves in each voxel. The results imply two important facts. First, it shows that the sLFO time shift information alone (delay map), applied to even arbitrary (but identical) timecourses, will result in a number of distinct ICs in ICA, which are highly similar to well-known RSNs. In other words, the actual temporal signal of each voxel is irrelevant in producing these ICs resembling RSNs. This finding is surprising. It is commonly believed that all the neuronal fluctuations happening during the resting state are reflected in the temporal BOLD signals of the voxels. If so, the neuronal activity in different RSNs will result in different temporal BOLD signals between networks, and ICA will identify these neuronal signals and corresponding brain regions (i.e., networks). Here we offer an alternative way to obtain these “networks” with synthetic data, unrelated to the neuronal fluctuations. We show that since ICA is sensitive to the temporal shifts in the signals, it identifies these symmetric brain regions in distinct groups even when the only variable is the temporal shift in each voxel. Second, the reason that these meaningful symmetric RSNs were identified (e.g., all six symmetric RSNs in the Beckmann template), instead of random symmetric patterns, is probably that the cerebral vasculature associated with these networks (e.g., visual network) is likely denser or more complicated in these areas due to their functional importance (Collins et al., 2010). For instance, the vascular density in visual cortex is likely higher due to the importance and complexity of the visual function. These vascular features are reflected in the hemodynamic delay map (i.e., more uniform time delay in these regions), thus leading to the detection of the “networks.” This explains the consistency between the RSNs and specific neuroanatomical system. However, even if this is the case, the identified networks still reflect the local vascular anatomy as result of million years of evolution, rather than spontaneous neuronal fluctuations.

These arguments are not confined to the results of ICA. As we show in Figure 6, similar RSNs (i.e., the DMN) were also detected from both real and synthetic group data derived through seed analysis. It is not as surprising; seed analysis is even more sensitive to the temporal shifts of the data, since the method itself is based on searching regions with high temporal correlations with the seed voxel. The regions with the same time delays of the sLFOs will be detected as “highly correlated,” while the regions with different time delays are not “correlated,” even when all these temporal signals are the same oscillations, differing only in phase (i.e., synthetic data).

These sLFOs-related ICs share many of the characteristics of the RSNs commonly found in resting state studies. For example, the detected networks are robust. Since the basic blood vasculature is stable (which would lead to a stable delay map), it is not surprising that many RSNs are consistently found in many states, including sleep (Deco et al., 2014), sedation (Greicius et al., 2008), and even vegetative state (Vanhaudenhuyse et al., 2010), in many conditions (i.e., depression, schizophrenia), and even in other mammals (Rilling et al., 2007; Zhang et al., 2010a). The small variations found in these networks from different states and populations could also be explained by the corresponding vascular changes, for example, schizophrenia patients are known to have altered blood flow in certain brain regions (Cohen et al., 1995) relative to neurotypical subjects, which would presumably lead to changes in their vascular delay map. Moreover, the existence of stable vascular “networks” offers an intriguing explanation to some somewhat surprising results in the literature. For example many split brain patients maintain a surprisingly high level of interhemispheric coherence as seen in RSN studies (Uddin et al., 2008). While there is a convincing argument to be made for information transfer over extracallosal neuronal pathways, it is also true that callosal surgery is unlikely to disturb the symmetry of vascular arrival times; some of the preservation of bilateral networks may reflect the contribution of these “vascular networks.” Recently, Christen et al. found temporally shifted BOLD signals in the resting state data of Moyamoya patients and disturbed RSN patterns (Christen et al., 2015). These are likely caused by the blocked arteries at the base of the brain, which directly support the argument of vascular network and its effects on RSN detections.

**Figure 7:**
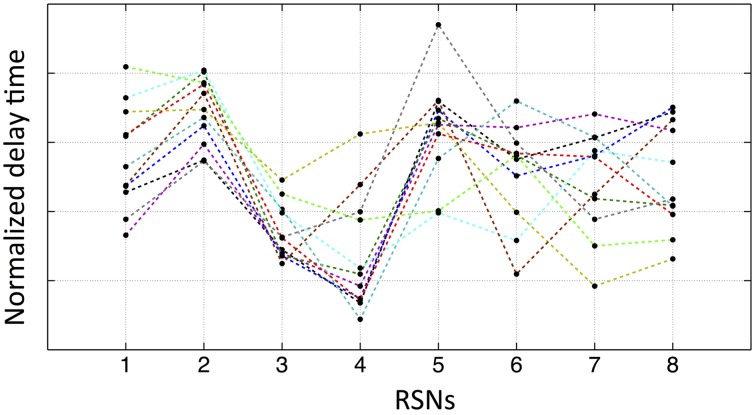
**Normalized average temporal delays of each networks (networks 1–8 in the same order as in Figure 5) for each participant**. Each color line represents one participant by connecting the average temporal delays of eight networks from this participant.

We have demonstrated that purely vascular signals can give rise to spatial networks which match common RSNs. If this is the case, there should be relative delays between these networks that are determined by their relative locations on the vascular “tree.” While there will be individual variation, we would expect that some networks should consistently be “ahead” of certain other networks due to the shorter vascular distance to the arterial root. To test this, for each subject, we extracted the averaged temporal delay for each RSN from the delay map. The results are shown in Figure 7, where each colored line represents one subject's normalized delay trace connecting the eight RSNs' averaged temporal delays (i.e., the networks 1–8 are in the same order as shown in Figure 5). The delay time matrix (with standard deviation of each delay value) is given in the Supplementary Material (for the purpose of display, the error bars are not shown in Figure 7, while the similar graph with error bars is given Supplementary Figure 3 with offsets). From these results, we ran a paired *t*-test (two tails with 5% confidence level) on each pair of networks to assess their relative temporal relationship. Even when Bonferroni correction was applied to account for the 56 comparisons performed, there were still a number of significant relationships. For example, network 3 (the auditory network) is consistently ahead of network 2 (the lateral visual network) (as shown in Figure 7). In general, networks 3 and 4 (the sensory–motor network) are “early” vascular networks, networks 2 and 5 (DMN) are late ones. Networks 1, 6, 7, and 8 are roughly in the middle with no clear order. These results are strong indications of internal robust temporal relationships between networks. If the delay maps were the results of spurious correlation calculation, we should not find any consistent relationships among networks from 11 subjects. The possible explanations for some networks having clear ranking, while others do not, could be: (1) some networks might lie on the same vascular branch (passage) with clear sequential relationship, while the others are on different ones with similar distance to the root. (2) Some networks are more vascular, while the others are more neuronal. More studies are needed to clarify the issue.

### Interpretation of the results

In this manuscript, we demonstrate that signals depending only on vascular time delays can generate ICs similar to RSNs through ICA, and offer the argument that these non-neuronal ICs might share some features as commonly attributed to RSNs. To be clear, we are not denying the existence of the neuronal networks. There is ample evidence from other imaging modalities to support the neuronal origin of RSNs. These imaging modalities include the technologies that assess the neuronal activation indirectly, such as positron emission tomography (PET) and arterial spin labeling (ASL) MRI, and those measuring neuronal activation directly, such as electroencephalogram (EEG) and magnetoencephalography (MEG).

PET is sensitive to brain metabolism. The DMN was initially identified by O15-PET to be the areas showing increase in oxygen extraction fraction (i.e., decrease in brain activity) during cognitive tasks (Raichle et al., 2001). Recent FDG-PET studies confirm the existence of many networks, including visual, auditory and motor networks through glucose utilization. However, they were not able to find the DMN (Di et al., 2012). Similar RSNs were identified by ASL that is sensitive to the blood flow changes (Biswal et al., 1997; Zou et al., 2009; Viviani et al., 2011). Like BOLD fMRI, these methods are not a direct measurement of neuronal activities, thus they are still prone to the influence of underlying vascular structure.

However, there is direct evidence of neuronal networks from EEG/MEG studies, which measure the neuronal signal directly. In concurrent EEG/fMRI studies, the data has shown correlations between cortical electrical activity (measured by electroencephalogram) and certain RSNs' slow BOLD oscillations in resting state studies (Martinez-Montes et al., 2004; Hiltunen et al., 2014). Moreover, an independent MEG study has shown good spatial agreement with the RSNs found by fMRI (Brookes et al., 2011). These results offer the direct support of the existence of RSNs of neuronal origin.

One explanation for our finding is the coexistent of both vascular and neuronal networks. There is no reason to believe that vascular networks are fully independent of neuronal function. Their role is to support neurons, thus their structure and development is likely decided by some neuronal factors, such as neuronal density (Collins et al., 2010). As result, the vascular networks are likely overlapping or partially overlapping with the neuronal networks that they serve. Bright et al., found spatially overlapping DMNs of neuronal and physiological origins in task and resting state data (Bright and Murphy, 2014), while Birn et al. found the network similar to DMN using the respiratory regressor (Birn et al., 2006). This evidence supports the hypothesis of overlapping vascular and neuronal networks. Based on these assumptions, we believe that the RSNs detected by ICA or seed analysis in fMRI are likely a mixture of both types of networks. Unfortunately, PET and MEG, especially MEG, have much lower spatial resolution than fMRI, so at the present time their independent RSN results (which are most likely purely neuronal) cannot be used to directly interpret the fMRI results. However, in the recent study on RSNs' changes with age, Balsters et al. used EEG-informed fMRI analyses to separate networks of neuronal origin from those of non-neuronal origin (Balsters et al., 2013).

In terms of signals, the contribution of spontaneous neuronal fluctuations to BOLD fMRI is hard to identify due to large physiological oscillations, and lack of a neuronal task regressor in resting state. Based on our studies, the sLFOs (identified by their time lagged correlation with sLFOs signals) appear to explain ~30% variance in the BOLD signal in gray matter, while respiration and cardiac pulsation could contribute another 10–15% (Chang et al., 2009). There are other physiological processes including motion, CO_2_ level, etc. Even with these non-neuronal factors, neuronal fluctuation likely still contributes a considerable amount of signal. To accurately quantify it, however, it is critical to identify and remove various physiological fluctuations.

### Future studies

Effective denoising is critical in RSN studies. There are many denoising methods proposed to remove different physiological signals from the resting state BOLD (Murphy et al., 2013), however for the most part these have treated low frequency oscillations as a epiphenomenon of cardiac and respiratory fluctuations, or as an aliasing artifact. We argue that the sLFO signal must be considered directly as an independent physiological noise source. First, we need to understand the sLFO signal and measure it accurately. We have shown that the sLFOs measured by NIRS at periphery, or derived recursively from the BOLD, are essentially the same traveling signal (Tong et al., 2012), which is different from LFOs derived from models using the respiratory and cardiac signals (Birn et al., 2008; Chang et al., 2009). We have found that sLFOs are major contributors to the BOLD. However, these signals are poorly understood (Sassaroli et al., 2012). The literature attributes them to vasomotion and blood pressure wave (Nilsson and Aalkjaer, 2003), or simply describes them as “Mayer waves” (Julien, 2006), however, no consensus on their origin has been reached.

Second, our results show any denoising method developed needs to incorporate temporal information into the denoising procedure. Many physiological signals such as sLFOs, respiration, and cardiac pulsations, travel with different speeds inside the blood vessels throughout the body. They will arrive at different locations at different times. Using a static regressor, they cannot be effectively removed from the BOLD signals. Our method (Riptide) does characterize the temporal shifts of sLFOs so that they can be removed from the BOLD (Frederick et al., 2012); we continue to refine this method, and other denoising methods also include this type of temporal information (Chang et al., 2009).

As we discussed earlier, multimodality imaging techniques have great advantages, especially when they include EEG or MEG, which measure the neuronal activation directly. However, the experimenter must take care in performing these studies—many multimodality studies probing the spatial or temporal coherence of RSNs have treated the networks derived from fMRI as “the standard” in these comparisons. However, we argue that it is equally important to investigate the spatial and temporal differences, which might elucidate the difference between vascular and neuronal networks.

## Conclusion

We believe that this study has two important implications for resting state connectivity analysis. First, we have demonstrated that ICA methods (as well as seed analysis) are sensitive to temporally shifted BOLD signals, and as a result, different “networks” may be identified by ICA even if the only difference between these networks are the temporal shifts in the BOLD. Second, we have shown the possibility that some RSNs can be derived from non-neuronal signals, such as the temporally shifted sLFOs, which are prominent in resting state BOLD fMRI data. This study indicates the importance of fully assessing and removing these sLFOs. Otherwise, the apparent strength and extent of neuronal RSNs will be strongly biased by the hemodynamic information.

### Conflict of interest statement

The authors declare that the research was conducted in the absence of any commercial or financial relationships that could be construed as a potential conflict of interest.

## Supplementary material

Supplementary Figure 1 **Regressors derived from the recursive procedure are plotted in groups (at 3TR = 1.2 s intervals instead of 1 TR) for comparison**. Similarities between regressors, especially neighboring regressors are obvious, while progressive changes can also be observed.

Supplementary Figure 2 **A maximum *z*-statistic map (A) and sLFO delay map (B) of a single subject different from the one shown in Figures 3, 4**.

Supplementary Figure 3 **Normalized average temporal delays of each networks (networks 1–8 in the same order as in Figure 5) for each participant with error bars representing the standard deviations calculated from each network**.
