# Supplementary figures and images for: Can apparent resting state connectivity arise from systemic fluctuations?
Source: Front Hum Neurosci. 2015 May 15;9:285. doi: 10.3389/fnhum.2015.00285 (PMC4432665; doi:10.3389/fnhum.2015.00285)

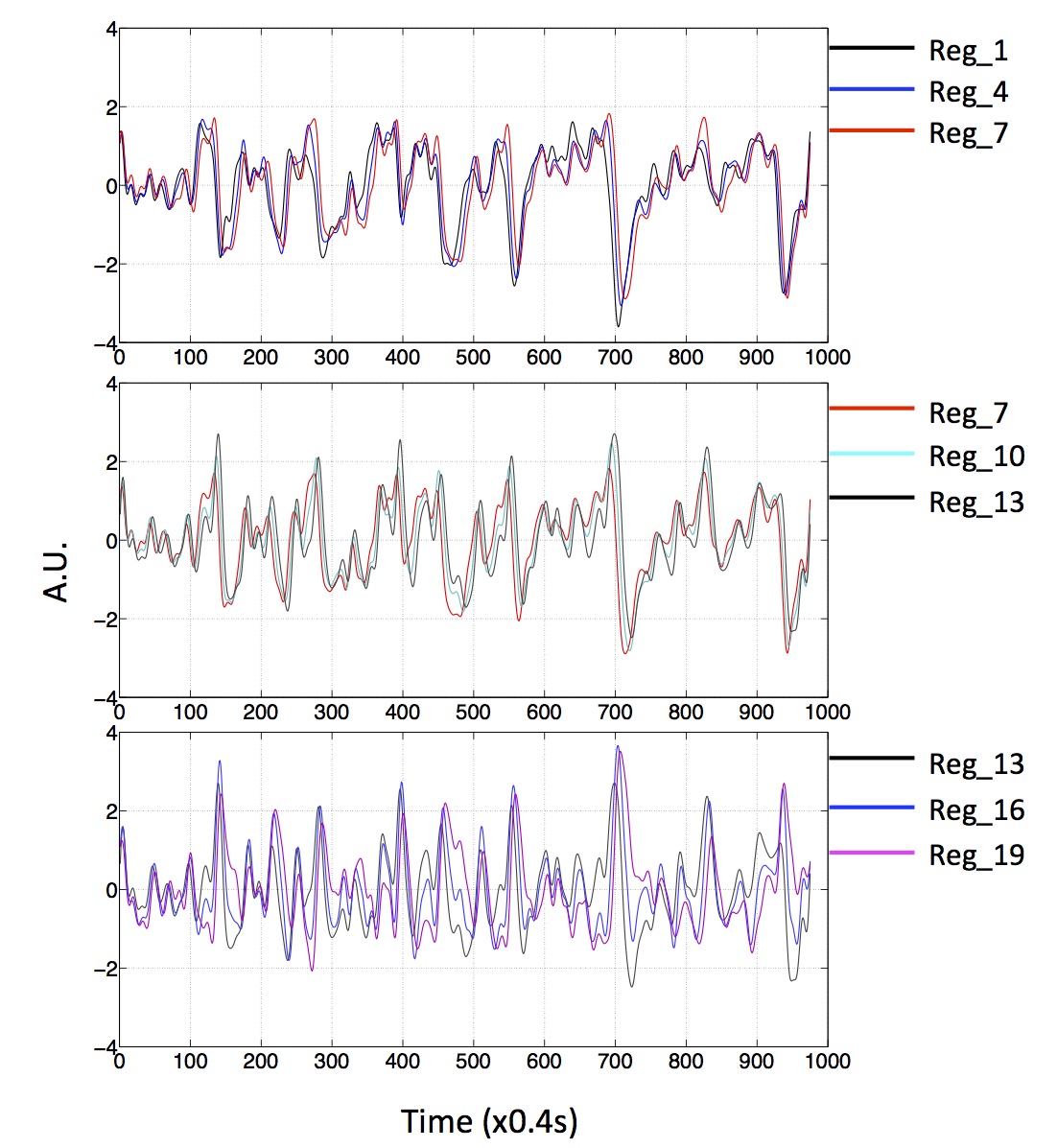

Supplement: Supplementary Figure 1 — Regressors derived from the recursive procedure are plotted in groups (at 3TR = 1.2 s intervals instead of 1 TR) for comparison. Similarities between regressors, especially neighboring regressors are obvious, while progressive changes can also be observed. [file Image1.JPEG]

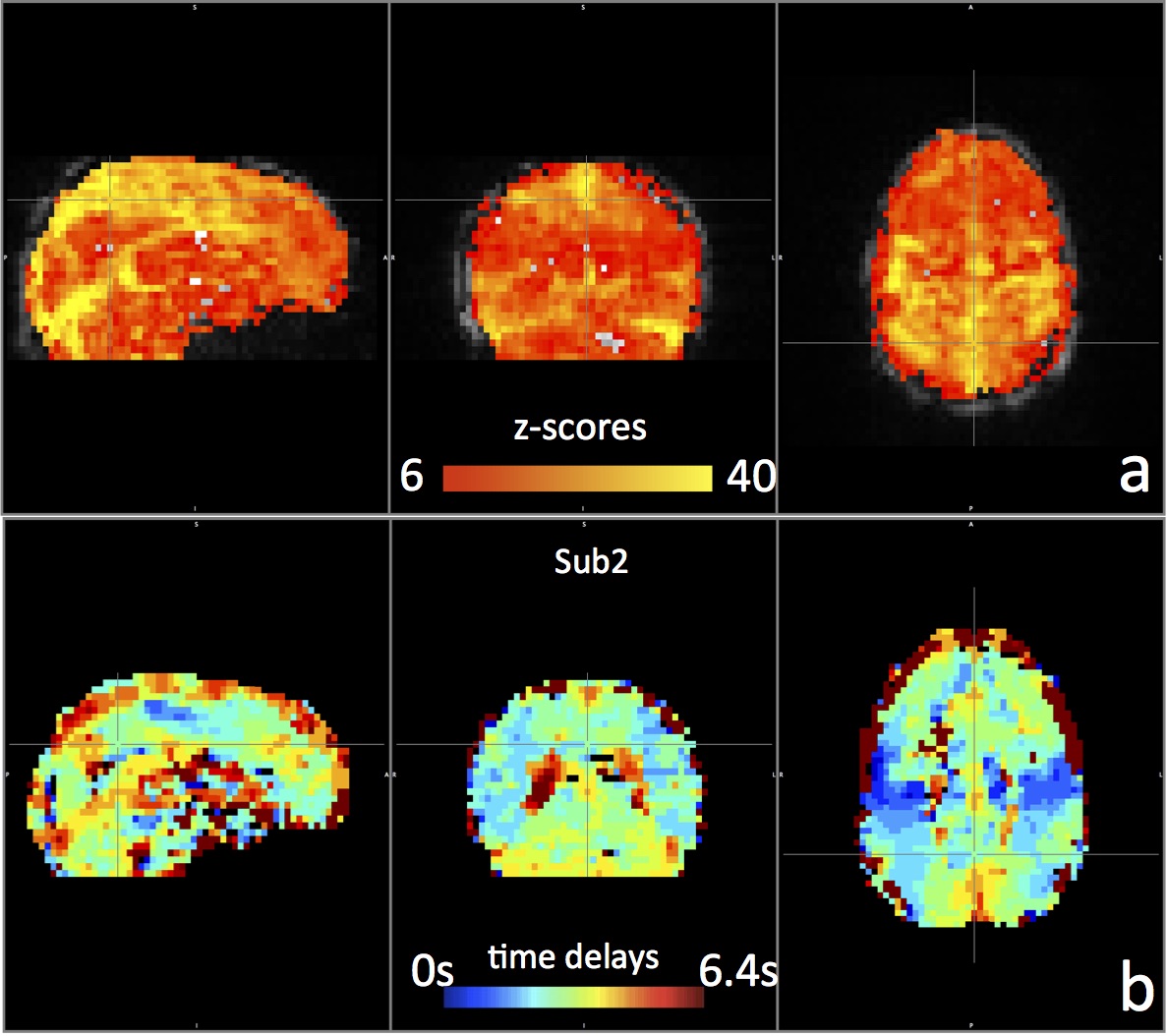

Supplement: Supplementary Figure 2 — A maximum z-statistic map (A) and sLFO delay map (B) of a single subject different from the one shown in Figures 3, 4. [file Image2.JPEG]

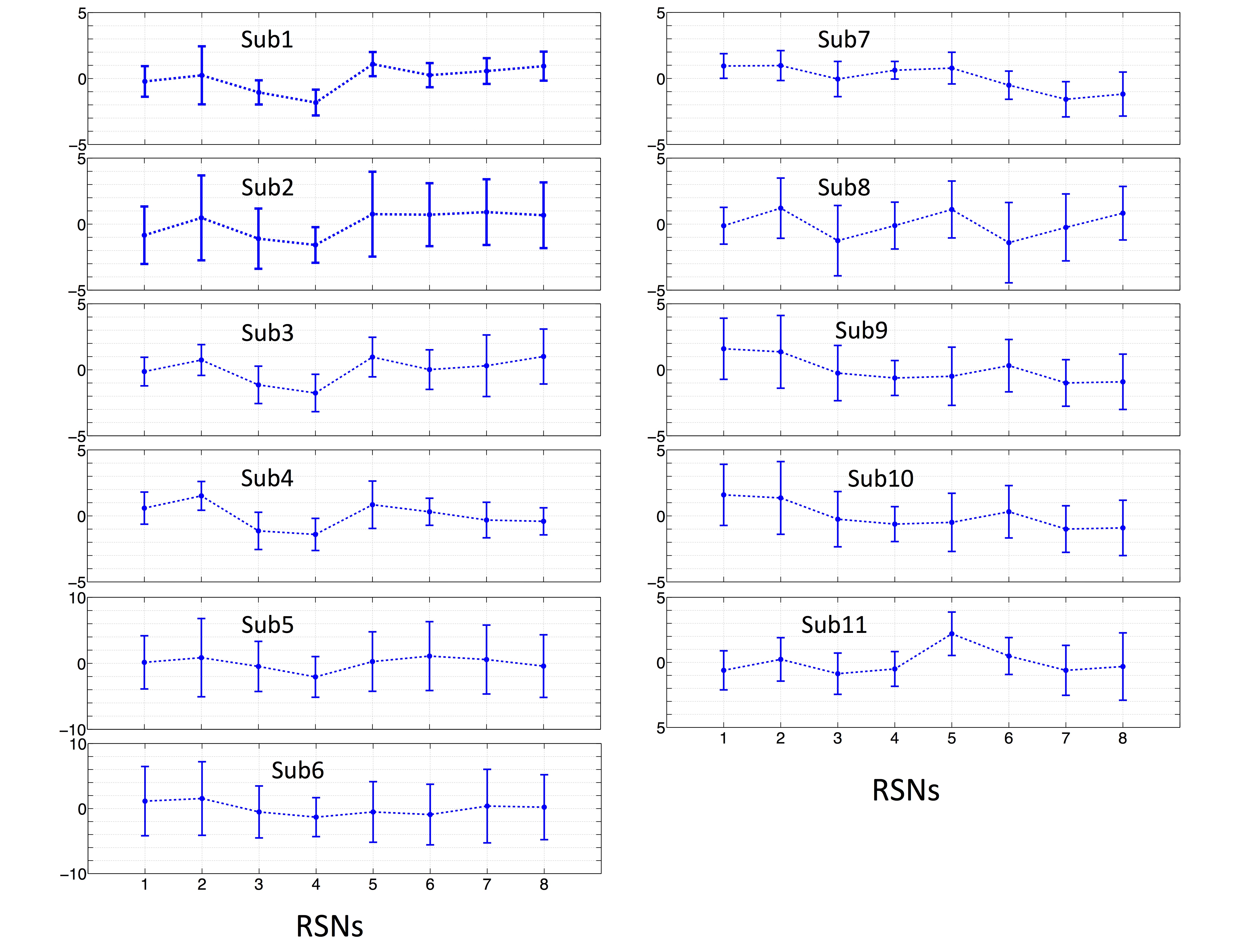

Supplement: Supplementary Figure 3 — Normalized average temporal delays of each networks (networks 1–8 in the same order as in Figure 5) for each participant with error bars representing the standard deviations calculated from each network. [file Image3.JPEG]
